# Effect of three different root canal sealants on human dental pulp stem cells

**DOI:** 10.1038/s41598-024-73232-y

**Published:** 2024-10-13

**Authors:** Ahmad Alfahlawy, Manar A. A. Selim, Hayam Y. Hassan

**Affiliations:** 1 Endodontic Department, Faculty of Dentistry, Suez Canal University, Ismailia, Egypt; 2 Oral Biology Department, Faculty of Dentistry, Suez Canal University, Ismailia, Egypt

**Keywords:** Cytotoxicity, Root canal sealer, GuttaFlow bioseal, Gutta-Percha, Well root St, AH-plus, Biological techniques, Stem cells

## Abstract

The cytotoxic effects of three root canal sealers with different bases on human dental pulp stem cells were assessed in this study using the 3-(4,5-dimethylthiazol-2-yl)-2,5-diphenyl tetrazolium bromide (MTT) test. The cytotoxic effects of three root canal sealers with different bases on human dental pulp stem cells (DPSCs) were assessed in this study using the 3-(4,5-dimethylthiazol-2-yl)-2,5-diphenyl tetrazolium bromide (MTT) test. The cytotoxicity of the sealers was tested after one, 4, and 7 d. Human dental pulp stem cell proliferation was concluded using an MTT assay. Cells not treated with sealer extract were used as controls. The absorption levels were measured using an Eliza spectrophotometer. P was set at 0.05 when the percentage of cell proliferation was matched between groups and observation times using one-way analysis of variance (ANOVA).During the second passage (P2), human dental pulp stem cells displayed a single morphological and phenotypic trait, with fibroblast morphology being the most common. There were no appreciable variations between the four groups after a day. There was a notable variation in the average percentage of cell proliferation between the groups after 4 and 7 days. The control group had the highest percentage, followed by the GuttaFlow Bioseal group, the Well Root St group, and the AH-Plus group, which had the lowest percentage. For every sealing group, after one day, the highest mean percentage of cell proliferation was recorded, followed by day four, and after day seven, the lowest mean percentage. The observation periods showed minimal cytotoxic effects of GuttaFlow Bioseal, whereas AH-Plus was the most cytotoxic to human dental pulp stem cells. The highest mean percentage of cell proliferation for all sealers was recorded on day one.

## Introduction

The root canal system must be sufficiently sealed in three dimensions to prevent bacteria, germs, and fluids from migrating from the coronal to the apical or vice versa, as well as to restore function and appearance, to prevent pain and infection from root canal fillings^1,2^.

Since it is commonly known that the materials used in root canal therapy might have less damage to the periradicular cell populations, less soluble materials should be utilized to avoid degradation by bodily fluids^3^.

There are other ways for the root canal to communicate with the surrounding tissues besides the apical foramen. The dentinal tubules, lateral and auxiliary canals, and the periodontal ligament and alveolar bone are among the many microscopic and macroscopic connections between the root canal and surrounding tissues^4^.

It is easy for tissue fluid to get within the root canal system, breaking down the sealer material and emitting different elements as a result. The periodontium and alveolar bone, which are nearby tissues, may then absorb these released components, leading to detrimental effects and localized periapical inflammatory reactions^5,6^.

The ability of the material to properly react biologically with the host tissue in a given application is one of the most important aspects of biocompatibility. This concept takes into account interactions between the drug, host, and anticipated function. The material will be biocompatible if these three elements work together harmoniously^7^.

Numerous techniques exist for measuring cytotoxicity, most of which use altered or tumor-derived cells as a model for the cell response. Nonetheless, there are other ways in which regular diploid cells are different from traditional or altered cells, like mitotic rate, growth regulation dependent on density, mitochondrial function, and medium^8^.

Research on stem cells is thought to cover a broad spectrum of science. Thanks to significant progress in stem cell biology, we can now repair damaged tissues caused by illnesses or traumas, opening up new treatment options^9,10^.

Many organs and tissues have been studied concerning adult stem cells. Including these tissues, the dental pulp is a soft connective tissue found inside the tooth’s pulp chamber and is regarded as a primary source of adult stem cells because, in comparison to other adult tissue sources, it has a higher cell content and requires less invasive methods for cell isolation^11,12^.

A root canal sealer constructed from calcium silicate has been called Well Root St. Late in 2015, a brand-new polydimethylsiloxane formulation called GuttaFlow Bioseal was introduced, combining gutta-percha powder and calcium silicate particles^13^. As a point of comparison, AH-Plus (Maillefer Dentsply, Switzerland), a root canal sealer that is frequently employed, was utilized^14^.

Three distinct root canal sealers Well Root St., GuttaFlow Bioseal, and AH-Plus—were tested for their cytotoxic effects on human dental pulp stem cells three times using the MTT assay. The study’s null hypothesis suggested no difference among the tested sealers.

## Materials and methods

### Sample size calculation

Based on the findings of a published study^15^, the sample size was determined using computer software known as (G* Power), producing a minimum of 84 samples (18 samples per group). The sample size was increased to (21 per group) (effect size = 0.46, Pooled SD = 183.76, and alpha (α) = 0.05) to account for samples that might have been lost.

## Randomization and blinding

Because the trial was double-blind, neither the data collector nor the data analyst who carried out the statistical analysis recognized which sealant was manipulated in the cytotoxic testing. Sealer example groups and subgroups were created using a coded number from the allocator. Utilizing computer software, random sequences (http://www.random.org/) were produced^16^.

## Teeth extraction

Ten unidentified maxillary premolar teeth from orthodontic patients were gently extracted with the aid of extraction forceps and a straight elevator to avoid fracturing the root. Every patient signed a written informed consent form. The teeth were extracted at the oral and maxillofacial surgery department in the Faculty of Dentistry, Suez Canal University. Each extracted tooth was separately dipped in sterile phosphate-buffered saline (PBS) accompanied with 5% penicillin–streptomycin, saved in an ice pack to avoid tissue damage, and directly transferred to the cell culture lab for sample processing. The teeth were kept in the solution for 15 min to eliminate any bacterial contamination^17^.

## Cell cultures preparation

### Preparing an aseptic environment

Laminar flow hood under aseptic conditions to ensure sterility. Ethanol (70%) was used to spray all the surfaces before use and allowed to dry. Sterilized glassware, plastic, and pipettes were used for tissue culture. The hood’s ultraviolet germicidal lamp was turned on for 15 min to sterilize the interior and contents before usage to prevent contamination during the experiment. Gloves were worn at all times and sprayed frequently with 70% ethanol to maintain sterility^18^.

## Dental pulp extirpation

The teeth were separated at the cementoenamel junction by a diamond bur on a low-speed micro-motor handpiece by sterilized saline as the coolant. The tissues were extirpated using a suitably sized sterilized endodontic barbed broach^20^.

## Stem cell isolation and culture

### Stem cell isolation

Dental pulp tissues were immersed in a 2 mg/mL collagenase solution and incubated in 5% CO_2_ at 37°C for 2h. Cell medium (Dulbecco’s modified Eagle’s medium (DMEM) + 10% fetal calf serum + 1% penicillin, neomycin, and streptomycin) was combined to stop the collagenase effect and transported to a conical tube. The tube was centrifuged at 1800 rpm for 2 min^21^.

## Cell seeding and observation

Before cell seeding, the cell medium (200 mL) achieved the desired cell seeding density (1 × 106 cells) was calculated. Cells were then placed into flasks with cell medium (DMEM + 10% fetal calf serum + 1% penicillin, neomycin, and streptomycin) and kept at 37°C and 5% CO_2_ in an incubator. The cells were microscopically observed every 24 h to check cell viability, foreign objects, bacteria, fungi, or cell morphology^21^.

## Medium exchange

The cells were cultured in an incubator with CO_2_. Since the culture medium’s nutrients were digested by the cells, a new medium was added to replace the one that had become nutrient-depleted and high in metabolites. Before the medium change, the cells were observed to confirm that the culture proceeded normally. The old medium was quickly replaced with a newly pre-warmed one to prevent the drying of the cells. We looked for any indications of damage to the cells. After taking pictures of the culture under a microscope to confirm that everything was going according to plan, the culture was put back into the incubator^22^.

## Passage

Once the cells started to proliferate, they were divided into new culture flasks before their current flask became full. This is termed "passage."

In this case, the cells that were grown in the culture flask were called "confluent." The cells were passaged when the area lodged by the cells reached nearly 70–80% of the flask.

Trypsin was utilized to separate the DPSCs because they attached to the walls of the culture flask as they became confluent, a phenomenon known as "contact inhibition," when cells came into contact with one another and believed that they no longer needed to proliferate. It was important to wash off the culture media containing calcium or magnesium ions with PBS, which does not include Ca2 + /Mg2 + , before application since trypsin action is hindered by these ions.

1 cc of trypsin per 25 cm2. After giving the flasks a little shake, they were kept in an incubator for ten minutes. Subsequently, the cells were observed under a microscope to confirm that they had separated from the base and were floating. Subsequently, the cells were suspended in a small amount of cell media (100–200μl) to inactivate the trypsin, and the cells were counted using a hemocytometer and light microscope. The cells were ready for use when a number (1 × 106) was reached^23^.

## Determination of cell viability

Using the trypan blue exclusion assay, cell survival was assessed at the start and finish of the culture in the manner described below:

- After trypsinizing the cells, a full medium (DMEM plus 10% FBS) was used to prepare the cell suspension.
- In a test tube, a combination of 100 µL cell suspension and 100 µL 0.4% trypan blue solution was made. When trypan blue leaks into cells with damaged cell membranes, it becomes visible because it is a membrane lipid-insoluble dye.
- After two minutes, a Pasteur pipette was used to take a drop of the mixture and put it in a hemocytometer chamber. Dead cells were blue and frequently had uneven borders, whereas viable cells were clear and looked like bright circles.
- Cell viability was estimated to be > 95% after different passages. Using a light microscope, all viable cells in the 1 mm four-corner squares were calculated.
- The total number of cells was counted using the following equation:
  1 $${\text{Average cell count }} = {\text{ all viable cells counted }}/{\text{of squares counted Cells}}/{\text{ml }} = {\text{ average cell counts dilution factor}}\;106$$

Total cells in the original solution = cells/ml original volume of fluid from which the sample was removed^24^.

## Identification of DPSCs

Dental pulp stem cells can form adherent colonies that physically resemble fibroblasts (colony-forming unit-fibroblast (CFU-F) when they are flasked at low cell densities in the presence of a medium supplied with the appropriate serum. These were spindle-shaped cells that multiplied in the culture media and were firmly adhered to the culture flask^25^. Tissue culture flasks containing 1 × 106 cells were inspected under a microscope, fixed with acetone/methanol (volume to volume), and stained with Giemsa dye to demonstrate the clonogenic capacity of the dental pulp stem cells. After counting cell colonies with more than 50 cells, the mean ± SD of the cells during the first and second passages (P1 and P2) was evaluated.

## Flow cytometric analysis of DPSCs

After trypsin-harvested DPSCs were three times washed away with PBS, aliquots of 1 × 106 cells were kept for 20 min on ice with phycoerythrin-conjugated monoclonal antibodies targeting CD34 and CD44. The cells were then washed three times with PBS provided with 1% Bovine Serum Albumin (BSA) and fixed for an entire night at 4°C in 1% formaldehyde. With CELLQUEST acquisition software from Becton Dickinson, 106 events were gathered for each sample on a Becton Dickinson FACS caliber flow cytometer, and Flow Jo software from Tree Star was used for analysis^26^.

## Sealer disc fabrication

The sealers (Table 1) were mixed as follows: Well Root St was offered by the manufacturer in a single syringe as all calcium silicate-based sealers, GuttaFlow Bioseal was provided in an auto mix syringe, and AH-Plus was provided in two separate tubes, base, and catalyst, which were added in a ratio 1:1 corresponding to the manufacturer’s instructions. Sixty-three sample discs of root canal sealers were constructed in sterile cylindrical Teflon molds has a height of 2 mm and a diameter of 5 mm. All samples of root canal sealers were mixed according to the manufacturer’s instructions and the discs were allowed to set at 37°C for 24 h before extraction. After complete setting, sealers were removed from Teflon blocks and samples were exposed to UV light for 24 h to prevent contamination and ensure sterility^14^.

**Table 1:** Sealers used in this study.

Material	Composition	Manufacturer
Well Root St Group A	Calcium silicate Zinc oxide Fillers	Vericom, South Korea
Guttaflow Bioseal Group B	Gutta percha powder Polydimethylsiloxane with nanometer-sized silver particles	Coltene Whaledent, Switzerland
AH-Plus Group C	Paste A: Epoxy resin, Calcium tungstate, Zirconium oxide, Aerosil, Iron oxide Paste B: Adamantane amine, N,N¢-Dibenzoyl-5-oxanonane diamine-1,9-TCD-diamine, Calcium tungstate, Zirconium oxide, Silicone oil, Aerosil	Dentsply Mallefer, Germany

## Elute preparation

Dulbecco’s modified Eagle’s medium (DMEM) was used as the extraction vehicle to extract the eluates of the various components in a sterile environment^27^. The process for extracting was as follows. The materials were kept for 24 h at 37°C in a humid environment with 5% CO_2_ in a 10 ml culture medium. After this time, the extraction medium was gathered and run through 0.22 mm filter paper.

## Cytotoxicity assay

To quantify cytotoxicity, a straightforward colorimetric technique created by Mosmann^28^ for testing cell survival and proliferation was modified. Just before usage, a 1 mg mL–1 solution of 3-(4,5-dimethylthiazol-2-yl)-2,5-diphenyl tetrazolium bromide (MTT) was made in full medium. The cells were sown in 96-well plates (1 × 106 cells per well) after being diluted in fresh complete media. Each group’s seven samples were tested throughout one, four, and seven days. Cells were cultured in a CO_2_ incubator for one, four, and seven days after being treated with different concentrations of sealers (200 mL/well). As controls, cells without sealer elute were employed.

Then each well received 50 mL of MTT dye. For four hours, the plates were kept in a CO_2_ incubator^29^. By eluting the dye with dimethyl sulfoxide (DMSO), optical density was ascertained, and a spectrophotometer was used to assess spectrophotometric absorbance at 550 nm. The following formula^28^ was used to measure the percentage of viable cells:

$$\% \;{\text{of}}\;{\text{cell}}\;{\text{viability}} = \frac{{{\text{Absorbance}}\;{\text{of}}\;{\text{sample}}}}{{{\text{Absorbance}}\;{\text{of}}\;{\text{control}}}} \times 100$$

## Statistical analysis

The Shapiro–Wilk test was determined if the data were normally allocated. The data is parametric and typically distributed. The descriptive statistics of the percentages of cell proliferation comprised the means and standard deviations. One-way ANOVA was used to examine the mean percentages of cell proliferation between groups and observation times. The mean percentage of cell growth varied significantly between the groups (P < 0.001) and between observation times (P < 0.001). The data was investigated using the (Statistical Package for Social Science, version 25).

## Results

### Cell culture results

**Fig. 1:**
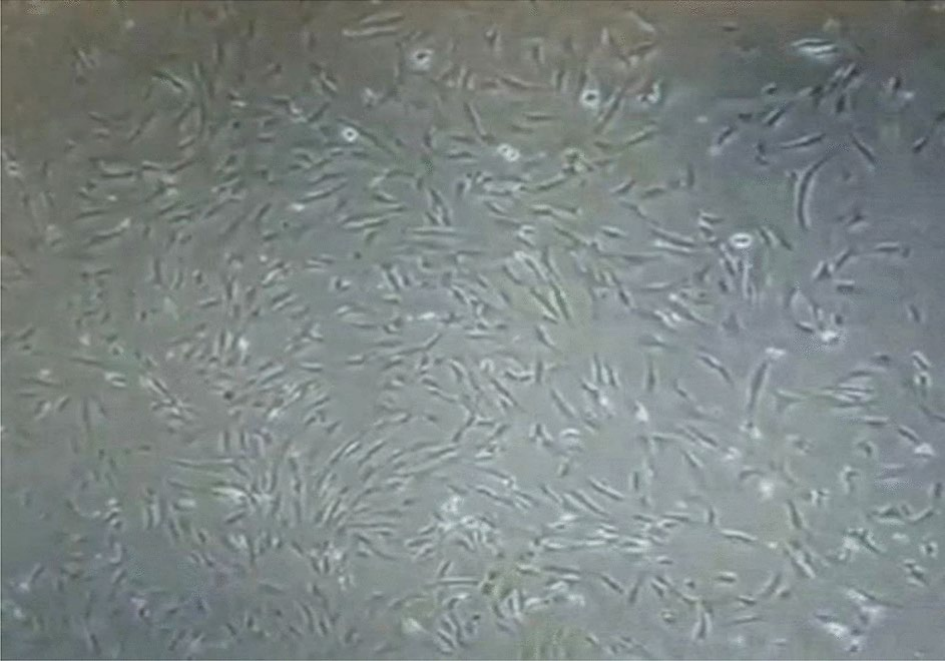
Microphotograph showing dental pulp stem cells under the light microscope at the end of P2 (20X).

Under the light microscope, during the 1st passage (P1), The cells proliferated and displayed a variety of morphological characteristics, including spindle, star, sperm-shaped, polygonal, or, in the case of the majority of the cells, fibroblast-like appearances. By the third day of the first passage, the cells had reached 50% confluence, and by the fifth day, they had reached 70% confluence. In the second section (P2), the cells displayed a single morphological and phenotypic characteristic, most of the cells had fibroblast morphology, evidence of colonies was observed, and cells maintained their morphology until the end of P2. The cells revealed a rapid growth rate, reaching 60% confluence on the 2nd day of P2 (Fig. 1).

## Identification of DPSCs

**Fig. 2:**
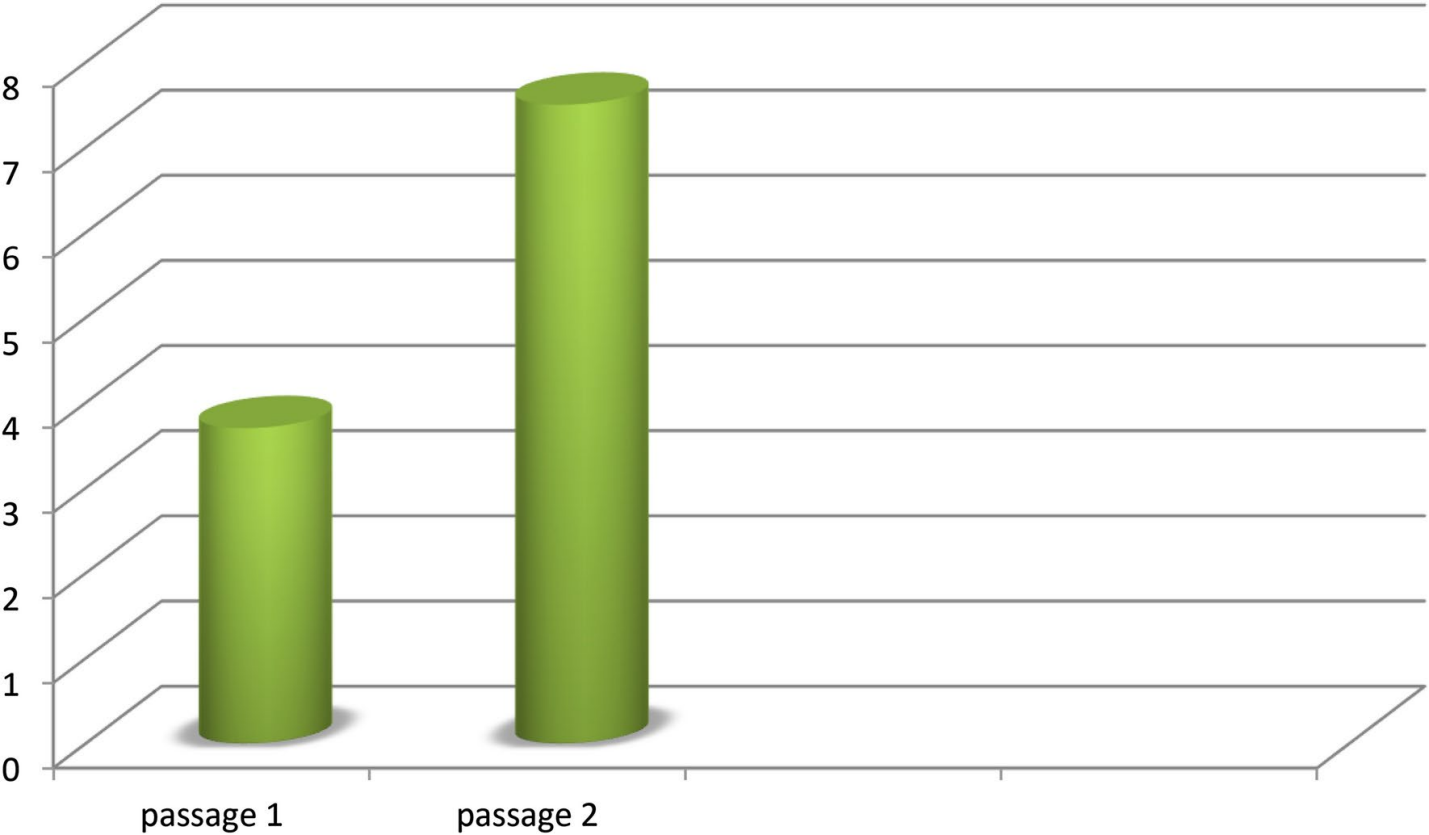
Chart showing the tendency of DPSCs to form colonies during P1 and P2. P2 cells showed more tendency to form colonies (*: *P* < 0.05).

Regarding the tendency of colony formation, dental pulp stem cells at both passages (P1) and (P2) presented a tendency to form colonies. There was a significant difference in P2 clonogenic potential (7.5 ± 3.1%) compared to that of P1 (3.7 ± 1.1%), P˂0.05, Fig. 2.

## Characterization of the isolated cells’ phenotype

**Fig. 3:**
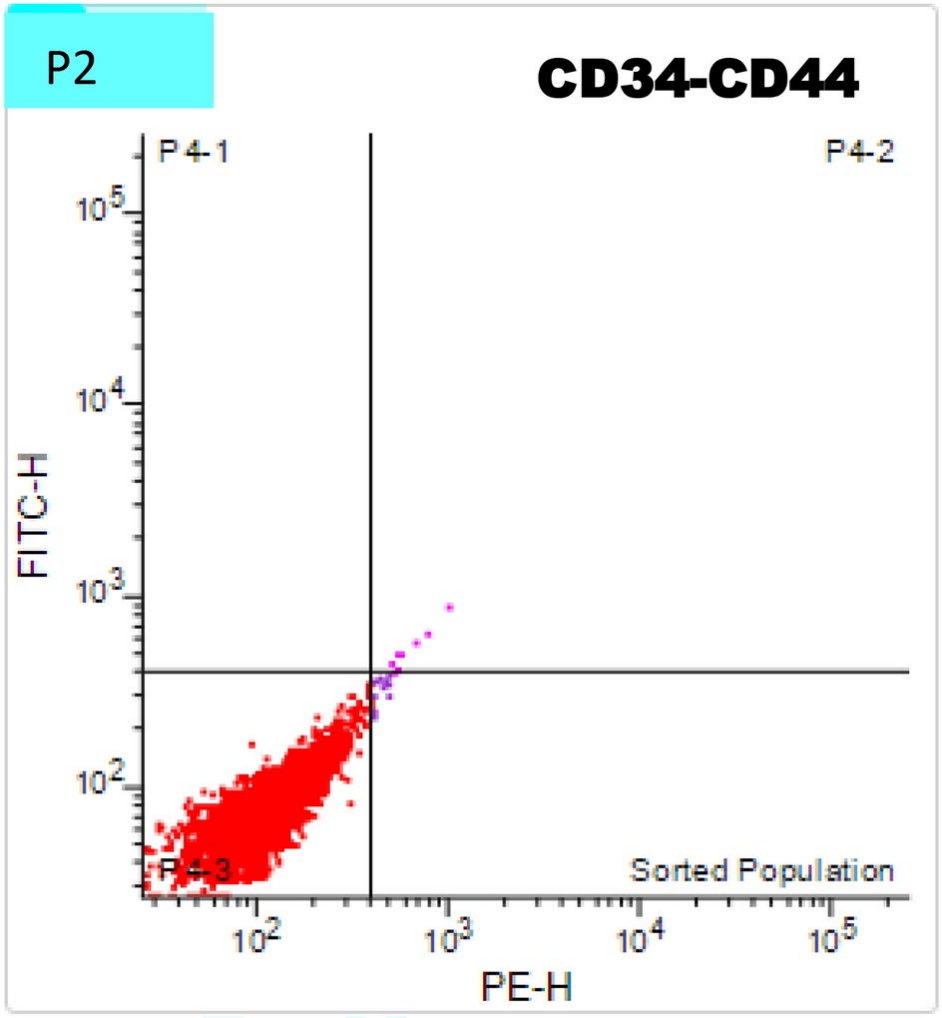
Chart showing the phenotypic analysis of the isolated pulp stem cells by flow cytometry, most of the cells expressed the mesenchymal stem cell marker CD44 and lacked that of hematopoietic stem cells CD34.

**Fig. 4:**
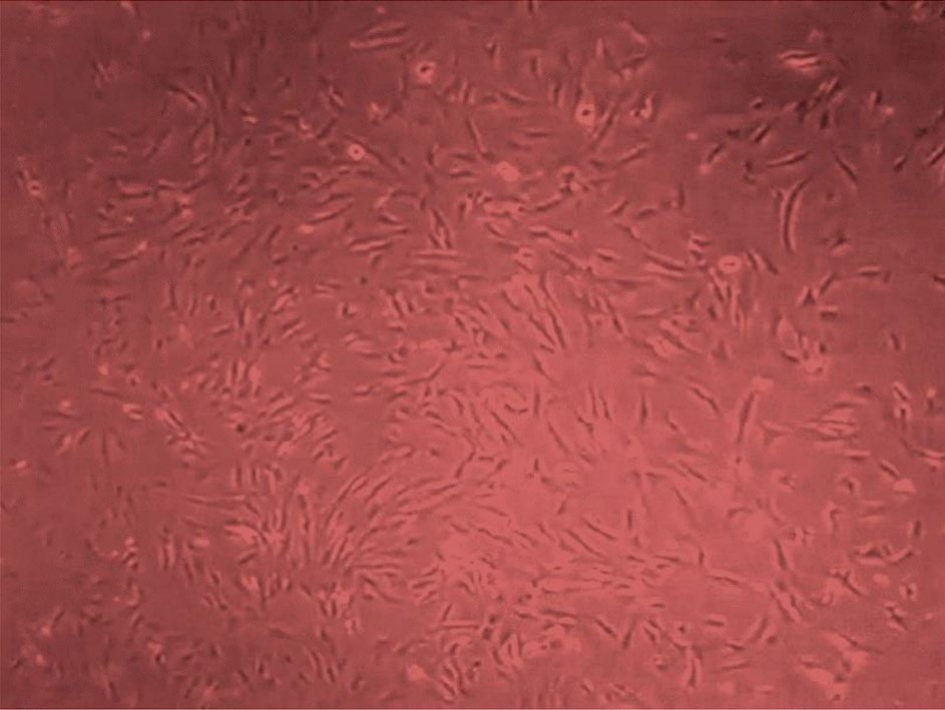
microphotograph showing phase contrast of DPSCs after P2 (20x).

70% of the isolated cells displayed the mesenchymal stem cell marker CD44 and did not have the hematopoietic stem cell marker CD34, according to flow cytometric examination of the cultured cells during P2, as shown in Figs. 3 and 4.

## MTT assay

- Comparison of mean percentage of cell proliferation between groups, Table 2

**Table 2:** Comparison of mean percentages of cell proliferation between groups and observation times.

	One day		4 days		7 days		Repeated measures ANOVA
	**X**	**SD**	**X**	**SD**	**X**	**SD**	
Control group	95.76 **A, a**	3.35	95.02 **A, a**	1.83	95.24**A, a**	2.69	**.96**
Group A	89.58 **A, a**	5.83	82.06 **B, C, b**	5.25	78.04 **B, C, b**	5.92	.001*
Group B	92.11 **A, a**	2.81	86.11 **B, b**	4.37	82.71 **B, b**	4.84	.008*
Group C	88.27**A, a**	6.76	79.21 **C, b**	5.58	72.52 **C, c**	2.57	< .001*
Repeated measures ANOVA	**.064**		< .001*		< .001*		

*p-value is significant at 5% level. Different lower-case letters in the same raw showed a significant difference between each 2 observation times (Bonferroni test, p < .05). Different upper-case letters in the same column indicate a significant difference between each 2 groups (Bonferroni test, p < .05). Same letters showed a non-significant difference between each 2 observation times or every 2 groups (Bonferroni test, p > .05), or every 2 groups (Bonferroni test, p > .05).

On the first day, there was no significant difference in the groups’ average percentage of cell growth. On days 4 and 7, there was a significant difference between the groups in the mean percentage of cell proliferation. Group B and Group A had lower mean percentages of cell growth than the control group. On the other hand, Group C had the lowest mean percentage. Furthermore, a noteworthy distinction was observed between every one of the two groups, except groups A, B, and C.

- Comparison of mean percentage of cell proliferation between observation times, Table 2

The control group’s mean percentage of proliferating cells did not differ significantly between observation times. On the other hand, the mean percentage of cell proliferation varied significantly between the other three groups. The maximum mean percentage of cell proliferation was recorded on day one, the lowest mean percentage on day seven, and the greatest mean percentage on day four. For groups A and B, there was a substantial difference between each of the two observation times, except day 4 and day 7.

## Discussion

This study included three different sealer categories used in endodontic practice: Well Root St, (a calcium silicate-based sealer); GuttaFlow Bioseal, (a silicone-based sealer); and AH-Plus, (an epoxy resin-based sealer). Calcium silicate is the main component in Well Root St sealer; it ionizes with water to form C-S–H gel and provides good cytocompatibility. These sealers’ high alkalinity may alter the surrounding conditions to assist the creation of hard tissues and obstruct osteoclastic activity in nearby tissues, both of which are favorable to healing^30,31^.

Late in 2015, a brand-new polydimethylsiloxane formulation called GuttaFlow Bioseal was introduced. It contains gutta-percha powder and calcium silicate particles.

By giving the tooth natural repair components like calcium and silicates, this bioactive substance enhances treatment outcomes and canal regeneration. Upon interaction with the liquids. The concept is quite straightforward: the new GuttaFlow Bioseal generates hydroxyapatite crystals on its surface upon curing, greatly enhancing adhesion and actively promoting dentine and bone tissue regeneration.

This would make it easier for professionals to give their patients long-lasting remedies. Previously, only MTA and bioglass showed the same healing qualities as GuttaFlow Bioseal; nevertheless, these materials required laborious handling and a protracted curing period. Innovative Bioseal, which is simple to use and cures in 12–16 min, removes these challenges. GuttaFlow Bioseal offers consistent and efficient results for endodontic practitioners, regardless of their level of experience^32^.

Despite the development of many methods and materials with various physicochemical and biological qualities, AH-Plus is still the most often utilized substance in filling procedures^33^.

Testing the possible negative impacts of materials can be done through animal tests, clinical investigations, and cell culture. Nevertheless, innovative cell culture techniques have been created to substitute animal models, enhance the comparability of data produced in various testing facilities, and save expenses and the usage of test animals by preventing redundant testing^34^.

Assessing the cytotoxicity of new endodontic materials is a crucial step towards getting them for therapeutic use. The main cellular models utilized in cytotoxicity studies are primary cell cultures^35,36^. Every one of these cell models has benefits and drawbacks. The high expense and unpredictability of basic cultures, as well as the genetic instability or physiological reactions of altered strains, are the fundamental causes of these drawbacks^37,38^.

As an alternative, stem cells are an excellent model for toxicity prediction because they are more applicable to the prediction of toxicity in humans and may be used to assess the effects of cytotoxicity on both cell viability and differentiation processes^39^. Notably, they are accessible from commercial sources and serve as a reliable in vitro model for human multipotent cell populations^40^.

As such, the cell type that can be selected for in vitro biocompatibility investigations is important to take into account. Because of their tight kinship with endodontic cement and sealers, immortalized human dental pulp stem cells were handled for this investigation^41,42^. Cell culture manipulation of human dental pulp stem cells can be carried out for a limited number of passages, with negligible effect on the cells^43^. One disadvantage of employing primary cell culture in research is that it must be utilized sparingly to prevent contamination or cell death^44^. For this reason, DPSCs were only used on day 7 of the study.

As previously mentioned, an enzymatic digestion technique was used to separate dental pulp stem cells. It may be because it takes longer for cells to migrate from the pulp in the explant approach that some research favors enzymatic digestion over the outgrowth technique^45^. Spath et al.^46^ have demonstrated that explant-derived cultures have improved proliferation and differentiation potential in comparison to those obtained by enzymatic digestion.

To enable the cultivated cells to stick to the adherent culture system, DPSCs were grown there. The system was then incubated at 37°C with 5% CO_2_ in the air. In stem cell research, this is the standard method. This approach is in line with earlier research conducted by Obeid et al.^48^ and Baldión et al.^47^.

Seven days after digestion of the pulp tissue, cell proliferation was observed. The cells had a fibroblast-like morphology with long cytoplasmic processes. Most cells through the 2nd passage showed a homogeneous fibroblast-like morphology. These findings were confirmed by Patil et al.^49^.

The trypan blue dye exclusion test was utilized to measure cell viability and concentration since it is the most widely used and straightforward technique^50^. It is based on the theory that live cells have intact cell membranes that prevent the absorption of certain dyes, including trypan blue. The findings showed that 95% of the cells were viable, with viable cells having clear cytoplasm and nonviable cells having blue cytoplasm^51^.

The ability of DPSCs to form adherent colonies with fibroblast-like morphology and the distinctive spindle-shaped cells were used to identify them. Using flow cytometry to characterize the DPSCs revealed that they were positive for CD44 and negative for CD34 surface markers, indicating that they were mesenchymal. This aligns with findings from additional research conducted by Pivoriuūnas et al.^52^ and Mehrabani et al.^53^.

The MTT assay is a popular method for assessing cytotoxicity and a well-regarded colorimetric assay for the quantitative evaluation of metabolically active cells. Finding out if living cells’ mitochondrial dehydrogenase enzymes can change yellow, soluble tetrazolium salt (MTT) into dark blue formazan crystals is the main objective of this research. This assay can be used to differentiate between living and dead cells since dead cells are unable to produce the vivid formazan result. The activity of the mitochondrial enzymes is directly correlated with the amount of formazan produced in a particular cell line. The MTT technique offers the following advantages: it can potentially reduce time and is precise, dependable, and simple to use^54,55^.

The MTT assay made it feasible to cultivate in small amounts and conduct sensitive evaluations, enabling the screening and testing of a wide range of compounds and fractions to determine their cytotoxicity. Yet, the number of cells and MTT decrease do not always correlate favorably. Above all, the MTT assay allows for the existence of live cells with low metabolic activity because it assesses metabolic activity rather than necessarily cell viability^56^.

In this work, the use of GuttaFlow Bioseal extracts increased the proliferation of DPSCs from pulp tissue grown in comparison to the use of Well Root St or AH-Plus. These findings showed that the components can have a direct impact on the cytotoxicity caused by sealers.

At all observation times, GuttaFlow Bioseal showed the highest proliferation percentage, whereas AH-Plus showed the lowest percentage. Well Root St was between them.

Over time, there was an increase in cytotoxicity and a decrease in the proliferation percentage within each group. The reason for this is that longer durations expose the medium’s contact surface to more of the root canal sealer or endodontic cement, which in turn produces more leaching molecules and increases the endodontic material’s cytotoxicity.

The elevated amine content of AH-Plus, which is utilized to shorten the setting period, could be the cause of its noticeably greater cytotoxicity^57^. Cytotoxicity may result from the release of bisphenol-A-diglycidyl ether, a mutagenic substance found in resin-based materials. Conversely, endodontic silicone-based sealers have demonstrated strong biocompatibility^58^.

According to Ju Kyung Lee et al.^59^, Well Root ST sealers gradually lost cell viability in fresh media, possibly as a result of their high pH when they were first introduced. Because calcium silicate-based sealers break down into calcium hydroxide when they come into touch with soft tissues, they have a high alkalinity^60^.

Elgendy et al.^61^ stated that Well-Root ST was associated with significantly the highest cell viability percentages, but, AH plus significantly showed less cell viability in comparison to (Ceraseal) calcium silicate-based root canal sealers.

Well-Root ST had a considerably higher proportion of viable cells. While AH-Plus exhibits a considerable increase in cell survival when compared to root canal sealers based on calcium silicate. Collado et al.^62^ reported that GuttaFlow Bioseal showed better cytocompatibility than GuttaFlow2, AH-Plus, and MTA Fillapex.

Pereira et al.^63^ recorded the promising MTT results obtained allowing us to conclude that GuttaFlow Bioseal is the least cytotoxic material studied. It features a higher adherence and proliferation of cells that are in contact with this material. The studies with these endodontic sealers also indicate that the cytotoxicity of these materials increases with time.

According to Claudio et al.^64^ BioRoot RCS and TotalFill BC sealers which are based on calcium silicate and include Well Root St. and AH-Plus did not exhibit any cytotoxic effects, at least not for the first 24 h, but not for the next 72. The other sealers were only slightly harmful, while AH-Plus was extremely toxic. Conversely, Joao et al.^65^ found that AH-Plus was extremely cytotoxic when it was first mixed and that its toxicity diminished over time.

## Conclusion

AH-Plus exhibited the highest cytotoxicity towards human dental pulp stem cells, whereas GuttaFlow Bioseal showed minimal cytotoxicity throughout the observation duration. For all sealers, the first day had the highest mean percentage of cell proliferation. More research is required to ascertain the physiological response to cytotoxic effects on periapical tissues over time, considering. It was found that human cells were not harmed by minor negative impacts on physical and sealing qualities. As a result, these innovative bioactive materials may lengthen tooth life, safeguard tooth roots, and improve the effectiveness of endodontic therapy. The limitations of this in vitro study suggested further preclinical studies to support advanced clinical applications and demonstrate the safety and efficacy of sealers. However, additional studies are necessary to clarify its mechanism of action. Furthermore, it was discovered that the same material had different effects on the fate of stem cells from different sources.

## Data Availability

The datasets generated and analyzed during this investigation are not publicly available due to (ownership of data); however, they are available from the corresponding author upon reasonable request.

## References

[1] Gutmann, J. L. Grossman’s endodontic practice-13^th^ Edition. *J. Conserv. Dent.: JCD* **19**(5), 494. 10.4103/0972-0707.190011 (2016).

[2] Johnson, W., Kulild, J. C. & Tay, F. Obturation of the cleaned and shaped root canal system. In *Cohen´s Pathway of the Pulp* 11th edn (eds Hargreaves, K. H. & Berman, L. H.) 280–323 (Elsevier, St. Louis, 2016).

[3] Granchi, D. et al. Endodontic cements induce alterations in the cell cycle of in vitro cultured osteoblasts. *Oral Surg. Oral Med. Oral Pathol. Oral Radiol. Endod.* **79**(3), 359–366. 10.1016/s1079-2104(05)80230-6 (1995). 7621013 10.1016/s1079-2104(05)80230-6

[4] de Pablo, O. V., Estevez, R., Péix Sánchez, M., Heilborn, C. & Cohenca, N. Root anatomy and canal configuration of the permanent mandibular first molar: a systematic review. *J. Endod.* **36**(12), 1919–1931. 10.1016/j.joen.2010.08.055 (2010). 21092807 10.1016/j.joen.2010.08.055

[5] Alfahlawy, A., Selim, M. A. A. & Hassan, H. Y. Biocompatibility of three different root canal sealers, experimental study. *BMC Oral Health* **23**(1), 715. 10.1186/s12903-023-03473-2 (2023). 37794396 10.1186/s12903-023-03473-2 PMC10552196

[6] Braga, J. M., Oliveira, R. R., de Castro, M. R., Vieira, L. Q. & Sobrinho, A. P. Assessment of the cytotoxicity of a mineral trioxide aggregate-based sealer concerning macrophage activity. *Dent. Traumatol.* **31**(5), 390–395. 10.1111/edt.12190 (2015). 26086068 10.1111/edt.12190

[7] Wataha, J. C. Principles of biocompatibility for dental practitioners. *J. Prosthet. Dent.* **86**(2), 203–209. 10.1067/mpr.2001.117056 (2001). 11514810 10.1067/mpr.2001.117056

[8] Huang, F. M., Tai, K. W., Chou, M. Y. & Chang, Y. C. Cytotoxicity of resin-, zinc oxide-eugenol-, and calcium hydroxide-based root canal sealers on human periodontal ligament cells and permanent V79 cells. *Int. Endod. J.* **35**(2), 153–158. 10.1046/j.1365-2591.2002.00459.x (2002). 11843970 10.1046/j.1365-2591.2002.00459.x

[9] Sylvester, K. G. & Longaker, M. T. Stem cells: review and update. *Arch. Surg.* **139**(1), 93–99. 10.1001/archsurg.139.1.93 (2004). 14718284 10.1001/archsurg.139.1.93

[10] Bianco, P., Robey, P. G. & Simmons, P. J. Mesenchymal stem cells: revisiting history, concepts, and assays. *Cell Stem Cell.* **2**(4), 313–319. 10.1016/j.stem.2008.03.002 (2008). 18397751 10.1016/j.stem.2008.03.002 PMC2613570

[11] d’Aquino, R. et al. Human dental pulp stem cells: from biology to clinical applications. *J. Exp. Zool. B Mol. Dev. Evol.* **312B**(5), 408–415. 10.1002/jez.b.21263 (2009). 19065566 10.1002/jez.b.21263

[12] Tirino, V., Paino, F., De Rosa, A. & Papaccio, G. Identification, isolation, characterization, and banking of human dental pulp stem cells. *Methods Mol. Biol.* **879**, 443–463. 10.1007/978-1-61779-815-3_26 (2012). 22610575 10.1007/978-1-61779-815-3_26

[13] Khalil, M. M., Abdelrahman, M. H. & El-Mallah, S. Bond strength and solubility of a novel polydimethylsiloxane-gutta-percha calcium silicate-containing root canal sealer. *Dent. Med. Probl.* **56**(2), 161–165. 10.17219/dmp/105626 (2019). 31274254 10.17219/dmp/105626

[14] Andriukaitiene, L. et al. The effect of smear layer removal on E. *faecalis* leakage and bond strength of four resin-based root canal sealers. *BMC Oral Health.* **18**(1), 213. 10.1186/s12903-018-0655-7 (2018). 30545332 10.1186/s12903-018-0655-7 PMC6293555

[15] Viola, N. V. et al. Biocompatibility of an experimental MTA sealer implanted in the rat subcutaneous: Quantitative and immunohistochemical evaluation. *J. Biomed. Mater. Res. B Appl. Biomater.* **100**(7), 1773–1781. 10.1002/jbm.b.32744 (2012). 22821748 10.1002/jbm.b.32744

[16] Pandis, N., Polychronopoulou, A. & Eliades, T. Randomization in clinical trials in orthodontics: Its significance in research design and methods to achieve it. *Eur. J. Orthod.* **33**(6), 684–690. 10.1093/ejo/cjq141 (2011). 21320892 10.1093/ejo/cjq141

[17] Raoof, M. *et al.* A modified efficient method for dental pulp stem cell isolation. *Dent. Res. J.* **11**(2), 244–250 (2014). PMC4052652 24932197

[18] Verma, A., Verma, M. & Singh, A. Animal tissue culture principles and applications. *Animal Biotechnol.*10.1016/B978-0-12-811710-1.00012-4 (2020).

[19] Kim, I. H. *et al.* In vivo evaluation of decellularized human tooth scaffold for dental tissue regeneration. *Appl. Sci. (Basel).* **11**(18), 8472. 10.3390/app11188472 (2021). 36003951 10.3390/app11188472 PMC9397400

[20] Leite, M. L. *et al.* Bioactivity effects of extracellular matrix proteins on apical papilla cells. *J Appl. Oral Sci.* **29**, e20210038. 10.1590/1678-7757-2021-0038 (2021). 34495108 10.1590/1678-7757-2021-0038 PMC8425894

[21] Ahmed, B., Ragab, M. H., Galhom, R. A. & Hassan, H. Y. Evaluation of Dental Pulp Stem Cells Behavior after Odontogenic Differentiation Induction by Three Different Bioactive Materials on Two Different Scaffolds. *BMC Oral Health.* **23**, 252. 10.1186/s12903-023-02975-3 (2023). 37127635 10.1186/s12903-023-02975-3 PMC10150498

[22] Inada, E. *et al.* PiggyBac transposon-mediated gene delivery efficiently generates stable transfectants derived from cultured primary human deciduous tooth dental pulp cells (HDDPCs) and HDDPC-derived iPS cells. *Int. J. Oral Sci.* **7**(3), 144–154. 10.1038/ijos.2015.18 (2015). 26208039 10.1038/ijos.2015.18 PMC4582557

[23] Konjhodzic-Prcic, A., Jakupovic, S., Hasic-Brankovic, L. & Vukovic, A. Evaluation of Biocompatibility of Root Canal Sealers on L929 Fibroblasts with Multiscan EX Spectrophotometer. *Acta Inform. Med.* **23**(3), 135–137. 10.5455/aim.2015.23.135-137 (2015). 26236077 10.5455/aim.2015.23.135-137 PMC4499286

[24] Katsares, V. *et al.* A rapid and accurate method for the stem cell viability evaluation: the case of the thawed umbilical cord blood. *Lab. Med.* **40**, 557–560. 10.1309/LMLE8BVHYWCT82CL (2005).

[25] Suchánek J, Browne KZ, Kleplová TS, Mazurová Y. Protocols for dental-related stem cells isolation, amplification and differentiation. Dental Stem Cells: Regenerative Potential, Humana Press, Cham. Pp 2016. 27–56 10.1007/978-3-319-33299-4_2

[26] Carvalho, P. P. *et al.* Use of animal protein-free products for passaging adherent human adipose-derived stromal/stem cells. *Cytotherapy.* **13**(5), 594–597. 10.3109/14653249.2010.544721 (2011). 21198335 10.3109/14653249.2010.544721

[27] Tsitrou, E. et al. Effect of extraction media and storage time on the elution of monomers from four contemporary resin composite materials. *Toxicol. Int.* **21**(1), 89–95. 10.4103/0971-6580.128811 (2014). 24748741 10.4103/0971-6580.128811 PMC3989922

[28] Mosmann, T. Rapid colorimetric assay for cellular growth and survival: application to proliferation and cytotoxicity assays. *J. Immunol. Methods.* **65**(1–2), 55–63. 10.1016/0022-1759(83)90303-4 (1983). 6606682 10.1016/0022-1759(83)90303-4

[29] Karapınar-Kazandağ, M. *et al.* Cytotoxicity of 5 endodontic sealers on L929 cell line and human dental pulp cells. *Int. Endod. J.* **44**(7), 626–634. 10.1111/j.1365-2591.2011.01863.x (2011). 21306404 10.1111/j.1365-2591.2011.01863.x

[30] Prati, C. & Gandolfi, M. G. Calcium silicate bioactive cement: Biological perspectives and clinical applications. *Dent. Mater.* **31**(4), 351–370. 10.1016/j.dental.2015.01.004 (2015). 25662204 10.1016/j.dental.2015.01.004

[31] Zhou, H. M. et al. In vitro cytotoxicity of calcium silicate-containing endodontic sealers. *J. Endod.* **41**(1), 56–61. 10.1016/j.joen.2014.09.012 (2015). 25442721 10.1016/j.joen.2014.09.012

[32] Gandolfi, M. G., Siboni, F. & Prati, C. Properties of a novel polysiloxane-guttapercha calcium silicate-bioglass-containing root canal sealer. *Dent. Mater.* **32**(5), 113–126. 10.1016/j.dental.2016.03.001 (2016). 10.1016/j.dental.2016.03.001 27037059

[33] Marin-Bauza, G. A. et al. Physicochemical properties of methacrylate resin-based root canal sealers. *J Endod.* **36**(9), 1531–1536. 10.1016/j.joen.2010.05.002 (2010). 20728722 10.1016/j.joen.2010.05.002

[34] Schmalz, G., Widbiller, M. & Galler, K. M. Material tissue interaction-from toxicity to tissue regeneration. *Oper Dent.* **41**(2), 117–131. 10.2341/15-249-BL (2016). 26645359 10.2341/15-249-BL

[35] Peters, O. A. Research that matters - biocompatibility and cytotoxicity screening. *Int. Endod. J.* **46**(3), 195–197. 10.1111/iej.12047 (2013). 23398041 10.1111/iej.12047

[36] da Silva, E. J. N. L., Zaia, A. A. & Peters, O. A. Cytocompatibility of calcium silicate-based sealers in a three-dimensional cell culture model. *Clin. Oral Investig.* **21**(5), 1531–1536. 10.1007/s00784-016-1918-9 (2017). 27460565 10.1007/s00784-016-1918-9

[37] da Silva, J. M. *et al.* Effectiveness and biological compatibility of different generations of dentin adhesives. *Clin. Oral Investig.* **18**(2), 607–613. 10.1007/s00784-013-1000-9 (2014). 23712822 10.1007/s00784-013-1000-9

[38] Abud, A. P. *et al.* The use of human adipose-derived stem cells-based cytotoxicity assay for acute toxicity test. *Regul. Toxicol. Pharmacol.* **73**(3), 992–998. 10.1016/j.yrtph.2015.09.015 (2015). 26382612 10.1016/j.yrtph.2015.09.015

[39] Hook, L. A. Stem cell technology for drug discovery and development. *Drug Discov. Today.* **17**(7–8), 336–342. 10.1016/j.drudis.2011.11.001 (2012). 22100998 10.1016/j.drudis.2011.11.001

[40] De-Deus, G. et al. Optimal cytocompatibility of a bioceramic nanoparticulate cement in primary human mesenchymal cells. *J. Endod.* **35**(10), 1387–1390. 10.1016/j.joen.2009.06.022 (2009). 19801236 10.1016/j.joen.2009.06.022

[41] Economides, N., Pantelidou, O., Kokkas, A. & Tziafas, D. Short-term periradicular tissue response to mineral trioxide aggregate (MTA) as root-end filling material. *Int. Endod. J.* **36**(1), 44–48. 10.1046/j.0143-2885.2003.00611.x (2003). 12656513 10.1046/j.0143-2885.2003.00611.x

[42] Yoshino, P., Nishiyama, C. K., Modena, K. C., Santos, C. F. & Sipert, C. R. In vitro cytotoxicity of white MTA, MTA Fillapex® and Portland cement on human periodontal ligament fibroblasts. *Braz. Dent. J.* **24**(2), 111–116. 10.1590/0103-6440201302115 (2013). 23780362 10.1590/0103-6440201302115

[43] Karimjee, C. K., Koka, S., Rallis, D. M. & Gound, T. G. Cellular toxicity of mineral trioxide aggregates mixed with an alternative delivery vehicle. *Oral Surg. Oral Med. Oral Pathol. Oral Radiol. Endod.* **102**(4), 115–120. 10.1016/j.tripleo.2005.12.020 (2006). 10.1016/j.tripleo.2005.12.020 16997085

[44] Huang, G. T., Shagramanova, K. & Chan, S. W. Formation of odontoblast-like cells from cultured human dental pulp cells on dentin in vitro. *J. Endod.* **32**(11), 1066–1073. 10.1016/j.joen.2006.05.009 (2006). 17055908 10.1016/j.joen.2006.05.009

[45] Lizier, N. F. *et al.* Scaling-up of dental pulp stem cells isolated from multiple niches. *PLoS One.* **7**(6), 39885. 10.1371/journal.pone.0039885 (2012). 10.1371/journal.pone.0039885 PMC3387222 22768154

[46] Spath, L. *et al.* Explant-derived human dental pulp stem cells enhance differentiation and proliferation potentials. *J. Cell Mol. Med.* **14**(6B), 1635–1644. 10.1111/j.1582-4934.2009.00848.x (2010). 19602052 10.1111/j.1582-4934.2009.00848.x PMC3829026

[47] Baldión, P. A., Velandia-Romero, M. L. & Castellanos, J. E. Odontoblast-like cells differentiated from dental pulp stem cells retain their phenotype after subcultivation. *Int. J. Cell Biol.* **2018**, 6853189. 10.1155/2018/6853189 (2018). 29670655 10.1155/2018/6853189 PMC5836425

[48] Obeid, M., Saber Sel, D., Ismael Ael, D. & Hassanien, E. Mesenchymal stem cells promote hard-tissue repair after direct pulp capping. *J. Endod.* **39**(5), 626–631. 10.1016/j.joen.2012.12.012 (2013). 23611380 10.1016/j.joen.2012.12.012

[49] Patil, V. R., Kharat, A. H., Kulkarni, D. G., Kheur, S. M. & Bhonde, R. R. Long term explant culture for harvesting homogeneous population of human dental pulp stem cells. *Cell Biol. Int.* **42**(12), 1602–1610. 10.1002/cbin.11065 (2018). 30353965 10.1002/cbin.11065

[50] Aslantürk, Ö. S. In vitro cytotoxicity and cell viability assays: principles, advantages, and disadvantages. *InTech UK* **2**, 64. 10.5772/intechopen.71923 (2018).

[51] Alobaid, A. S. et al. Cell count and differentiation potential of isolated stem cells from extracted third molars. *Int. J. Med. Dent.* **23**, 46–50 (2019).

[52] Pivoriuūnas, A. *et al.* Proteomic analysis of stromal cells derived from the dental pulp of human exfoliated deciduous teeth. *Stem cells Dev.* **19**(7), 1081–1093. 10.1089/scd.2009.0315 (2010). 19824824 10.1089/scd.2009.0315

[53] Mehrabani, D. *et al.* Growth kinetics and characterization of human dental pulp stem cells: Comparison between third molar and first premolar teeth. *J. Clin. Exp. Dent.* **9**(2), 172–177. 10.4317/jced.52824 (2017). 10.4317/jced.52824 PMC5303312 28210430

[54] Da Fonseca Roberti Garcia, L. et al. Transdentinal cytotoxicity of resin-based luting cements to pulp cells. *Clin. Oral Investig.* **20**, 1559–1566. 10.1007/s00784-015-1630-1 (2016). 26481234 10.1007/s00784-015-1630-1

[55] Silva, G. O. et al. Cytotoxicity and genotoxicity of natural resin-based experimental endodontic sealers. *Clin. Oral Investig.* **20**, 815–819. 10.1007/s00784-015-1567-4 (2016). 26319976 10.1007/s00784-015-1567-4

[56] Rodrıguez-Lozano, F. J. et al. Evaluation of cytocompatibility of calcium silicate-based endodontic sealers and their effects on the biological responses of mesenchymal dental stem cells. *Int. Endod. J.* **50**, 67–76. 10.1111/iej.12596 (2017). 26660310 10.1111/iej.12596

[57] Miletic, I. et al. The cytotoxicity of RoekoSeal and AH-Plus compared during different setting periods. *J. Endod.* **31**, 307–309. 10.1097/01.don.0000140570.95688.ee (2005). 15793391 10.1097/01.don.0000140570.95688.ee

[58] Mandal, P., Zhao, J., Sah, S. K., Huang, Y. & Liu, J. In vitro cytotoxicity of guttaflow 2 on human gingival fibroblasts. *J. Endod.* **40**, 1156–1159. 10.1016/j.joen.2014.01.025 (2014). 25069924 10.1016/j.joen.2014.01.025

[59] Lee, J. K., Kim, S., Lee, S., Kim, H. C. & Kim, E. In vitro comparison of biocompatibility of calcium silicate-based root canal sealers. *Materials* **12**, 2411. 10.3390/ma12152411 (2019). 31362338 10.3390/ma12152411 PMC6695985

[60] Lim, E. S. et al. Physical properties and biocompatibility of an injectable calcium-silicate-based root canal sealer: In vitro and in vivo study. *BMC Oral Health* **15**, 129. 10.1186/s12903-015-0112-9 (2015). 26490372 10.1186/s12903-015-0112-9 PMC4618726

[61] Elgendy, A. Y. A comparative analysis of cytotoxicity of three different root canal sealers. *Int. J. Dent. Res.* **6**(2), 33–38. 10.31254/dentistry.2021.6203 (2021).

[62] Collado-Gonzalez, M., Tomas-Catala, C. J., Onate-Sanchez, R. E., Moraleda, J. M. & Rodriguez-Lozano, F. J. Cytotoxicity of GuttaFlow bioseal, GuttaFlow2, MTA Fillapex, and AH-Plus on human periodontal ligament stem cells. *J. Endod.* **43**, 816–822. 10.1016/j.joen.2017.01.001 (2017). 28343929 10.1016/j.joen.2017.01.001

[63] Pereira HP, Oliveiros JM, Santos D, Sequeira C, Brites P, Coimbra MM. Endodontic sealers in dentistry - in vitro and in vivo cytotoxicity studies. J Oral Sci, 2016, 171–177. https://www.researchgate.net/publication/315692658

[64] Poggio, C., Riva, P., Chiesa, M., Colombo, M. & Pietrocola, G. Comparative cytotoxicity evaluation of eight root canal sealers. *J. Clin. Exp. Dent.* **9**(4), 574–578. 10.4317/jced.53724 (2017). 10.4317/jced.53724 PMC5410681 28469826

[65] Silva, E. J., Santos, C. C. & Zaia, A. A. Long-term cytotoxic effects of contemporary root canal sealers. *J. Appl. Oral Sci.* **21**(1), 43–47. 10.1590/1678-7757201302304 (2013). 23559111 10.1590/1678-7757201302304 PMC3881813

